# Nonoperative management of hip fractures in very frail elderly patients may lead to a predictable short survival as part of advance care planning

**DOI:** 10.1080/17453674.2021.1959155

**Published:** 2021-07-28

**Authors:** Hugo H Wijnen, Peter P Schmitz, Houda Es-Safraouy, Lian A Roovers, Diana G Taekema, Job L C Van Susante

**Affiliations:** a Department of Clinical Geriatrics, Rijnstate, Arnhem;;; b Department of Orthopedics, Rijnstate, Arnhem;;; c Clinical Research Department, Rijnstate, Arnhem, the Netherlands

## Abstract

Background and purpose — Surgical treatment is still the mainstay of care even in very frail elderly hip fracture patients. However, one may argue whether surgery is in the best interest of all patients. We elucidated mortality rates of nonoperative management (NOM) of a hip fracture after shared decision-making in a cohort of very frail elderly patients.

Patients and methods — Orthogeriatric patients (age > 70 years) admitted with a hip fracture between 2011 and 2019 were included. In the presence of fragility features the motivation for surgery or NOM was supported by advance care planning (ACP) and shared decision-making through geriatric assessment. Mortality rates after NOM were assessed and also presented for the remaining surgical group for reference.

Results — In 1,279 out of 3,467 patients, geriatric assessment was indicated and subsequently 1,188 (93%) had surgery versus 91 (7%) NOM. The motivation for NOM was based on patient and family preferences in only 20% of patients, medical grounds in 54%, and a combination of both in 26%. The 30-day and 1-year mortality in the frail NOM group was 87% and 99% respectively, whereas this was 7% and 28% in the surgery group. No statistical comparison between groups was performed due to profound bias by indication.

Interpretation — This study provides further insight into the predictable and high short-term mortality after NOM in carefully selected very frail elderly hip fracture patients. This information may help to consider NOM as an alternative treatment option to surgery when no significant gain from surgery is anticipated.

The incidence of hip fractures in frail patients is rising due to an increase in life expectancy and cumulative comorbidities (Kanis 2002, Johnell and Kanis 2006, Ferrucci et al. 2016). In particular, frail elderly patients experience a substantial decrease in quality of life and mobility in the 12 months after hip fracture surgery (Amarilla-Donoso et al. 2020). Deliberations whether to operate or not in these frail elderly patients are common in daily practice (Dunn et al. 2016, van der Zwaard et al. 2020, Rietjens et al. 2021). Surgery is still the mainstay of treatment because it results in a better mobility and survival (van de Ree et al. 2017, Berry et al. 2018) and nonoperative management (NOM) is often characterized by problematic after-treatment with substantial patient discomfort. One may argue, however, whether surgery is in the best interest of all patients. It may be that the time has come to re-evaluate to what extent frail patients should always be treated surgically.

Frailty is negatively associated with quality of life after a hip fracture and may require tailored treatment, especially in patients with a short life expectancy and anticipated postoperative functional decline (van de Ree et al. 2019, Kanters et al. 2020). A hip fracture is a life-threatening condition in these frail patients and as such may be considered an opportunity to discuss end-of-life care, personal goals, and the preference for surgery or NOM with their potential pros and cons (Dunn et al. 2016) This concept is also known as “advance care planning” (ACP) (Teno et al. 1994, Johnston et al. 2018).

The literature on life expectancy after nonoperative management (NOM) is limited. For example, 30-day and 1-year mortality rates after NOM ranging from 5–65% and 30–95% have been reported (Loggers et al. 2020). These wide ranges of early mortality rates reflect differences in patient characteristics and fracture types between studies and extrapolation of information from these studies towards shared decision-making in clinical practice is difficult.

There is increasing awareness that operative treatment of a hip fracture may not always be the best option in all patients and that in a subgroup of frail elderly patients NOM should be considered and may also be patients’ preference. For this purpose it is important to obtain valid information on what can be expected in terms of survival after NOM in a well-selected subgroup of very frail patients. In this way informed consent and shared decision-making on treatment options for fragility hip fractures can be improved. We elucidated mortality rates in a consecutive group of 91 out of 1,279 patients with a fragility hip fracture treated with NOM after ACP and shared decision-making. Survival for the remaining surgically treated patients is also presented for reference. 

## Patients and methods

### Study population

**Figure 1.**
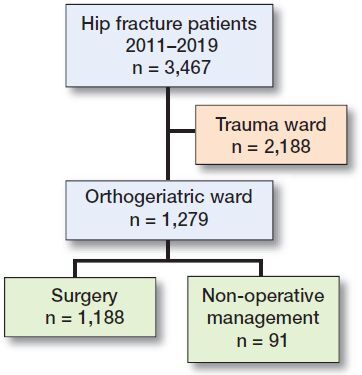
Flowchart of patient selection.

We performed a retrospective study to elucidate the course of mortality after NOM of a hip fracture. All consecutive hip fracture patients aged 70 years and older treated on the orthogeriatric ward were included between January 2011 and June 2019. Indication for admittance to the orthogeriatric ward was set by the orthogeriatric team after a comprehensive geriatric assessment on the somatic, psychiatric, functional, or social domain in order to identify fragility features. Patients with 1 or more fragility features were admitted to the orthogeriatric ward, whereas relatively vital and mostly younger patients were admitted to the surgical ward. The latter group of patients was not part of this study (Figure 1). Exclusion criteria were pathological hip fractures and periprosthetic hip fractures due to their impact on mortality.

The decision for surgery or NOM was made as a shared decision-making consultation with the patient and relatives. ACP was applied by mutually exploring treatment preferences, mobility, quality of life, goals in life, and survival in a comprehensive geriatric assessment (van der Zwaard et al. 2020). Surgery consisted of either hemiarthroplasty or internal fixation. The NOM approach focused on optimal comfort for the patient and mainly consisted of treatment of symptoms, in particular pain with analgesics. Patients returned to their homes or residential care facilities if feasible or were discharged to a nursing home with hospice care facilities.

### Study variables

The following parameters were retrospectively collected from the patient records: age, sex, Charlson Comorbidity Index (CCI), mobility, living situation, cognitive status (dementia), type of fracture, and type of surgery.

Reports from the shared-decision consultation resulting in a choice for NOM were reviewed. The reason for NOM was scored independently by 2 authors (HW and DT) as (1) based on patient and family preferences, (2) obvious medical grounds with unacceptable high perioperative risk, or (3) a combination of both. In the case of disagreement consensus was subsequently achieved by discussion between both authors.

If applicable, date of death was obtained from the medical records or from the Dutch Personal Records Database, which contains personal data of people who live in the Netherlands. Subsequently 30-day mortality and 1-year mortality rates were determined.

### Statistics

From a profound bias by indication together with a relatively small group on NOM no statistical analysis on differences between groups was performed.

### Ethics, funding, and potential conflicts of interest

Ethical approval for the study was granted by the Institutional Review Board (decision 2016-938). No funding was obtained and the authors have no conflicts of interest to declare. 

## Results

### Baseline characteristics (Table)

Between 2011 and 2019, 3,467 hip fracture patients were admitted to our hospital of which 1,279 frail elderly hip fracture patients (37%) were admitted to the orthogeriatric ward. From this study group 1,188 patients received surgical treatment whereas for 91 NOM was chosen. As such NOM was offered to 7.1% (91/1,279) of patients admitted to the orthogeriatric ward and to 2.6% (91/3,467) of all patients (Figure 1). As anticipated, the 1,188 patients in the surgery group were younger than the 91 patients in the NOM group—84 (SD 6.7) years versus 87 (SD 6.3) years, respectively. Pre-fracture mobility was better in patients who were operated on compared with those who were not, reflected by 45% of surgery patients being able to walk independently compared with 12% of patients in the NOM group. Further, dementia and living situation were different in the 2 groups, which is probably a reflection of the confounding bias by indication (NICE 2011).

**Table:** Patients’ characteristics at baseline. Values are count (%) unless otherwise specified

Population variable	Surgery n = 1,188	Nonoperative n = 91
Age (SD)	84 (6.7)	87
Sex (female)	877 (74)59	
Type of hip fracture		
Femoral neck	639 (54)	53
Trochanteric	444 (37)	35
Other	105 (9)	3
Charlson Comorbidity Index		
< 3	877 (74)	59
≥ 3	311 (26)	32
Living situation		
Independent	725 (61)	26
Sheltered care	149 (13)	19
Nursing home	200 (17)	46
Missing	114 (9)	
Dementia	317 (27)	48
Mobility		
Without assistance	511 (43)	9
Cane/walker	614 (52)	53
Wheelchair/bedridden	24 (2)	13
Missing	39 (3)	16

### Motivation for nonoperative management

In 18 of 91 patients the decision for NOM was clearly based on patient and family preference whereas medical grounds were not evident. In 49 patients NOM was almost completely dictated on medical grounds (comorbidity and high perioperative risk) and in 24 patients this was a combination of both.

### Mortality rates after nonoperative management and surgery

**Figure 2.**
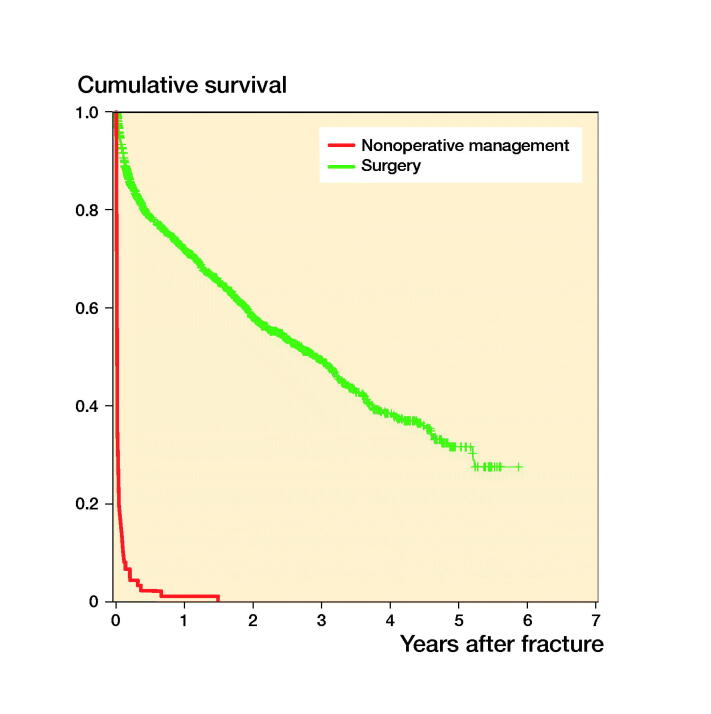
Kaplan–Meier survival analysis after nonoperative management (91 patients) and surgery (1,188 patients).

The 30-day and 1-year mortality rates in the frail NOM group were 87% and 99%, respectively (Figure 2). Of the 91 patients in the NOM group only 21 survived after 2 weeks, 12 were alive after 30 days, and only 3 after 3 months. Mean survival was 0.7 months in the nonoperatively managed patients.

The 30-day and 1-year mortality rates in the surgery group were 7% and 28%, respectively (Figure 2). Mean survival was 36 months in the surgically managed patients.  

## Discussion

This study was primarily conducted to provide more insight into the course of NOM in a selected group of very frail elderly hip fracture patients in whom ACP with patients and relatives had resulted in a shared decision not to operate to prolong life. The 30-day and 1-year mortality rates in this group were 87% and 99%, respectively. In contrast the reference 30-day and 1-year mortality rates in the surgery group were 7% and 28%, respectively. The choice for NOM was at least partly based on patient or family preference in almost half of these patients (46%), whereas the other half was treated nonoperatively mainly based on medical grounds (54%).

We found a very high 30-day mortality of 87% in nonoperatively managed hip fracture patients. These findings differ from other previously published studies with a 30-day mortality ranging from 5% to 65% (Chlebeck et al. 2019, Loggers et al. 2020). A recent review has already pointed out that these large differences in mortality rates are most probably caused by differences in patient characteristics such as pre-fracture mobility, prevalence of dementia, and living situation (Loggers et al. 2020). The clinical dilemma whether to decide for NOM and subsequent palliative care mainly applies to very frail elderly hip fracture patients. As such, it should be noted that the nonoperatively managed patients in our study were typically identified on the basis of their frailty with a low functional pre-fracture mobility who have not much to gain from surgery. In contrast, patients from available studies so far were less frail and had a better pre-fracture mobility, resulting in a treatment focus on active mobilization to regain function and prolong life (Loggers et al. 2020). For example, Berry et al. (2018) found a lower mortality rate of 54% at 6 months in a retrospective cohort study of 468 nonoperatively treated nursing home patients with advanced dementia and a hip fracture and Hossain et al. (2009) reported a mortality rate of 19% at 1 month in a retrospective cohort study of 21 nonoperatively treated patients. Again, we feel this can be explained by a different inclusion of patients for NOM. For example, Berry et al. also included patients with pelvic fractures and palliative care was initiated in only 34% of the patients, whereas in our study this accounted for all patients (Berry et al. 2018). In turn, Hossain et al. seemed to have used a different selection of patients as well, as a substantial number of non-displaced and impacted femoral neck fractures were included in which case NOM is an established approach to achieve healing of the fracture (Hossain et al. 2009). Again, this is an important confounder because nonoperative fracture treatment in non-displaced femoral neck fractures is entirely different from the palliative care approach in our study.

The choice of NOM in very frail elderly patients with a hip fracture is delicate and ethical, cultural, and legal issues apply, which can be different for countries. We believe that there is an increasing awareness that, in spite of the fact that—in general—surgery increases survival and the chances of regaining mobility, not all patients prefer surgical treatment. Instead, we should aim to improve recognition of this small group of very frail patients who have not much to gain from surgery and often do not wish to prolong life. This phenomenon is also well illustrated by a recent article of the Dutch association for medical doctors in which half of the nursing home patients indicated a preference for NOM above surgery should they be admitted to hospital with a hip fracture, irrespective of possible shorter survival (Stavenuiter et al. 2018). A retrospective evaluation of the reason for NOM in our study confirmed that patient preference is an important reason for NOM in almost half of the patients.

A thorough decision-making process is essential to recognize this specific group of very frail elderly patients in which NOM of a hip fracture may be considered. Frail elderly patients require ACP where multiple comorbidities, short life expectancy, anticipated postoperative functional decline, and low quality of life are discussed, together with patient preference personal goals and end-of-life wishes (van de Ree et al. 2019, Kanters et al. 2020). In this ACP the frail patients, their relatives, and healthcare professionals participate and decide together on NOM or surgery. The concept of patient-centered tailored treatment, ACP, was first conceptualized in the United States (Teno et al. 1994) with the purpose to receive medical care consistent with one’s preferences (Dunn et al. 2016, Johnston et al. 2018, Rietjens et al. 2021). ACP is increasingly integrated in daily care for frail elderly patients with a hip fracture in European countries, although there is still room for improvement (Evans et al. 2013).

This study has its limitations. The most important limitation is the recognized “confounding bias by indication” because the decision for NOM is typically made in a subgroup of frail patients with more comorbidities, higher age, lesser mobility, and dementia. In spite of the fact that these confounders can be corrected for in statistical models, we feel that this would oversimplify the problem and that profound bias would remain. One may argue whether this confounding is truly relevant with regards to the interpretation of the results. We decided to simply present the survival curves for NOM and surgery as reference, because it is beyond the scope of this study to compare mortality rates in NOM and operative treatment of hip fractures in elderly patients. Moreover, this study aimed to gain insight into the course of NOM with palliative care so that this option can be incorporated in balanced (shared) decision-making. Perhaps more importantly, from the mortality observed in the NOM group it appears that a rapid decline can be expected. Second, the inclusion of patients was limited to those patients admitted to the orthogeriatric ward, also indicating selection bias. However, these frail elderly patients in particular, selected by a comprehensive geriatric assessment, reflect the population of concern in the clinical dilemma of NOM of hip fractures. We feel that this clear selection bias may also reflect the high 30-day and 1-year mortality rates encountered in this study as compared with the available literature.

Besides limitations, important strengths also apply. In this study a rather homogeneous group of 91 very frail hip fracture patients were treated nonoperatively. This provided important information on the course of treatment and mortality, which is lacking in the available literature with a smaller number of patients and more importantly inhomogeneous fragility features (van de Ree et al. 2017). This information is important as it can be applied directly towards the clinical setting where we encounter the dilemma of whether surgery is indeed in the best interests of and in accordance with the preference of a patient with a fragility hip fracture.

In conclusion, this study revealed a predictable short survival after NOM of hip fractures in a group of very frail elderly patients where prolonged survival was not considered to be the primary goal of treatment. These results may reassure clinicians, patients, and their relatives that NOM can be regarded as a relevant treatment option with a predictable outcome in very frail hip fracture patients. In the case of limited life expectancy, information regarding nonoperative supportive management and well-managed pain in a palliative setting might help patients and their families to come to a well-founded decision in line with their wishes, goals, and end-of-life expectations. Therefore, in our opinion, NOM should gain more attention and ACP should be part of standardized preoperative hip fracture care in the frailest patients.
